# External Iliac Artery-Appendicular Fistula due to Antegrade Unusual Migration of K-Wire from Hip to Pelvis: An Unreported Complication

**DOI:** 10.1155/2015/207078

**Published:** 2015-06-03

**Authors:** Nagmani Singh, Chakra Raj Pandey, Bhaskar Raj Pant, Uttam Krishna Shrestha, Biraj Bista

**Affiliations:** ^1^Department of Orthopedic Surgery, Grande International Hospital, Kathmandu 44600, Nepal; ^2^Department of Vascular Surgery, Grande International Hospital, Kathmandu 44600, Nepal; ^3^Department of Radiology, Grande International Hospital, Kathmandu 44600, Nepal

## Abstract

*Background*. K-wires are thought to be extremely safe implants and complications as a result of direct insertion or migration are very rare. Complications may be life-threatening in some instances where migration results in injury to vital organs. We report one such case where antegrade migration of K-wire from the hip resulted in injury to external iliac artery and formation of external iliac artery-appendicular fistula. No such complication due to migration has ever been reported in the literature. *Case Description*. A 15-year-old boy presented with lower abdominal pain, right lower limb swelling and pain, inability to walk, and rectal bleeding for 1 month after 2 K-wires had been inserted in his right hip joint for treatment of slipped capital femoral epiphysis the previous year. On investigation, he was diagnosed to have external iliac artery-appendicular fistula for which he was surgically treated. *Clinical Relevance*. Antegrade migration of K-wire from hip joint may lead to life-threatening injuries which can be minimized by bending the end of the K-wire, keeping the tip protruding outside the skin wherever possible and by early removal of K-wire once its purpose has been achieved.

## 1. Introduction

Since its development by Martin Kirschner in 1909 [1], K-wires, which were initially used for providing skeletal tractions, are now one of the most commonly used implants in orthopedic surgeries for various purposes. In expert hands, K-wires can be used as an instrument as well as implant of choice in various orthopedic surgeries. However, when used improperly they can result in life-threatening complications like in our case. We hereby report a case of unusual K-wire migration in a 15-year-old boy from hip joint to pelvis leading to external iliac artery-appendicular/colonic fistula followed by rectal bleeding and subsequent appendicular rupture and peritonitis leading to septicemic shock.

## 2. Case Report

**Figure 1:**
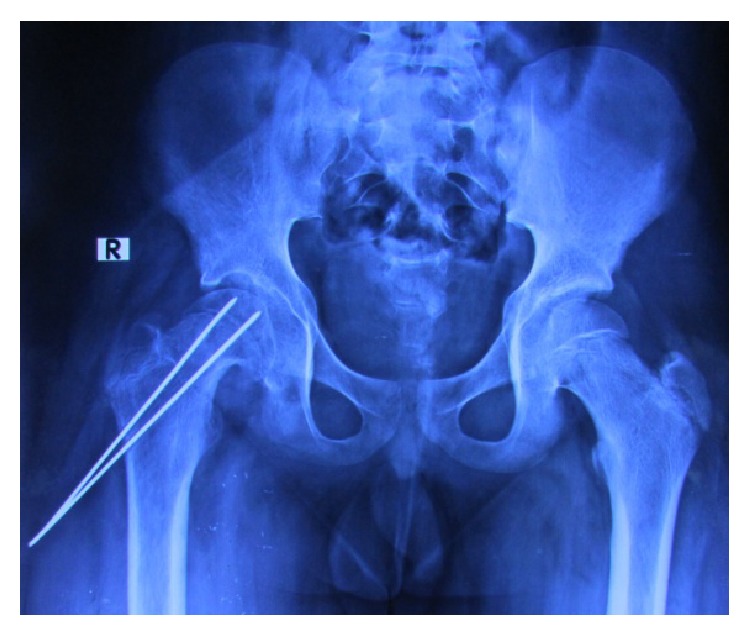
Postoperative radiograph showing K-wire fixation of the slipped capital femoral epiphysis at first surgery one year prior to presentation.

A 15-year-old boy was referred to us from another center with complaints of lower abdominal pain, right lower limb swelling and pain, inability to walk, and rectal bleeding for 1 month. Two surgeries, 7 days and 5 days before presentation, were performed recently to remove K-wires inserted in the same hip one year back for slipped capital femoral epiphysis (SCFE). He sustained SCFE following a fall while practicing karate. The fixation was done with 2 K-wires which were buried under the skin during that surgery and the tips of the wires were not bent (Figure 1).

**Figure 2:**
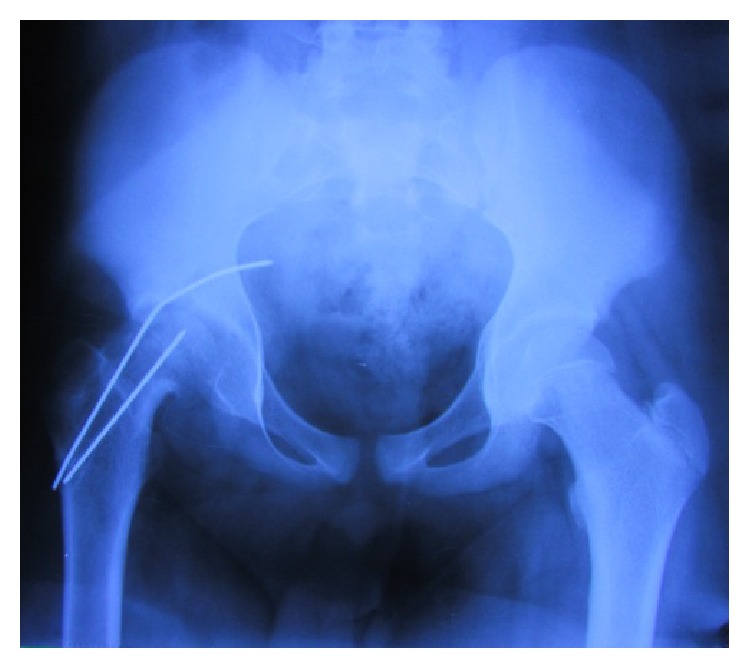
Postoperative radiograph shows intrapelvic migration of K-wire with remaining broken part of K-wire.

The postoperative period was uneventful and the patient was able to perform regular activities 12 weeks postoperatively. He developed the abovementioned complaints one month prior to presentation for which he was taken to the center where first surgery was done. During this surgery, one of the K-wires broke and another could not be retrieved through the hip incision (Figure 2).

**Figure 3:**
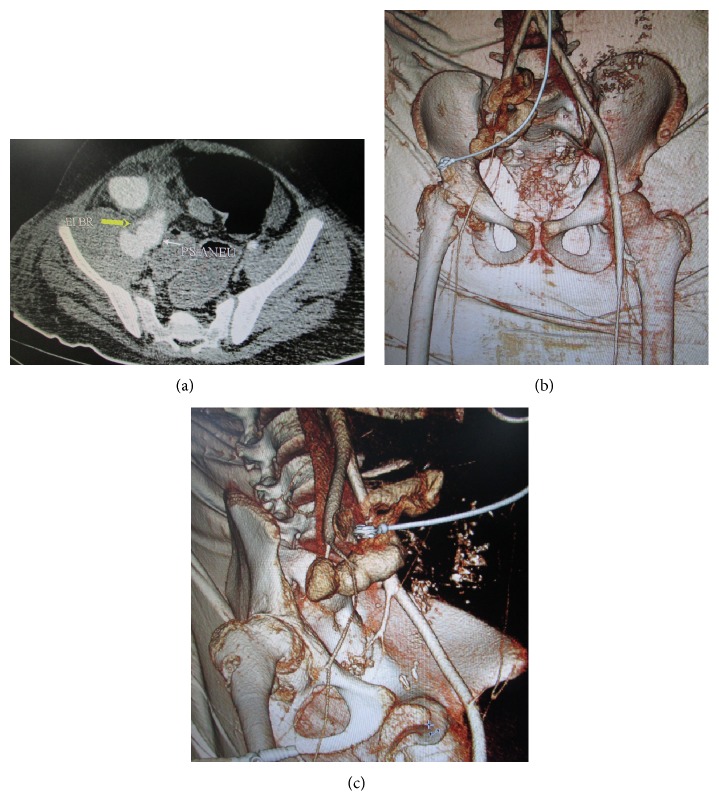
(a) Axial computerised tomography (CT) angiography image showing a branch of external iliac artery (EI BR) feeding the pseudoaneurysm (PS ANEU). (b) and (c) 3D reconstruction CT angiography images showing branch of the external iliac artery feeding the large bowel loops.

After surgery, the patient's condition deteriorated and he was brought to our center. At the time of presentation to us, the patient had high grade fever, tachycardia, weak pulse, unilateral right lower limb edema, and anxious look. On examination, a sutured wound in the right hip region and laparotomy wound were present. Rectal examination revealed fresh blood and bowel sounds were decreased. Movements of hip joint were restricted and extremely painful. Blood investigations revealed profound anemia, leukocytosis, and hypoalbuminemia. CT angiography revealed large pseudoaneurysm with a surrounding hematoma in the region of iliacus muscle, which was seen to be in direct communication with the caecum and large bowel loops as shown in Figures 3(a), 3(b), and 3(c).

**Figure 4:**
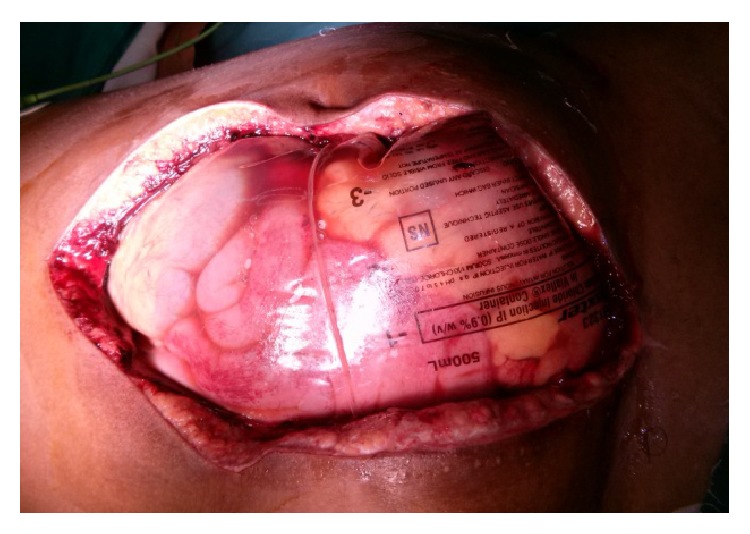
Shows Laparotomy wound which was packed because of massive bowel edema.

**Figure 5:**
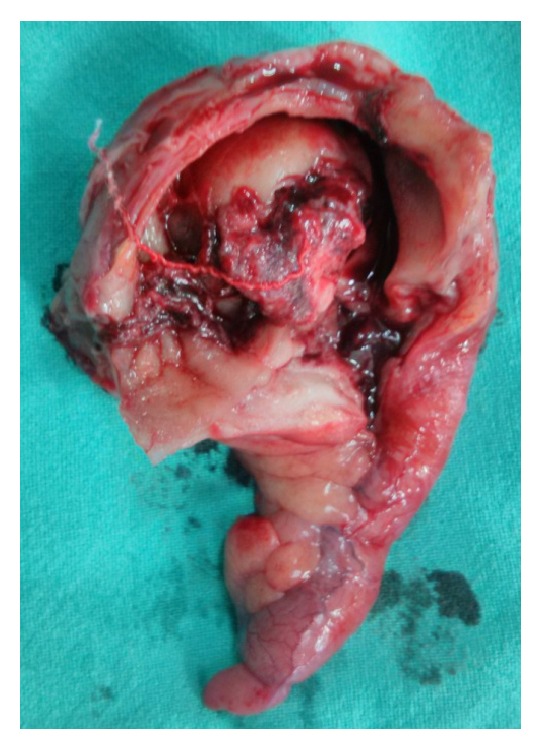
Resected part of the large bowel with appendix and caecum.

There was active contrast extravasation into pseudoaneurysm and large bowel loops. Direct communication of a branch of the external iliac artery into pseudoaneurysm was seen. Hematoma in the right thigh was also noted. There was reduced flow in the rest of the arterial system supplying lower limb. No abnormalities of the venous system in the right lower limb were seen. On CT scan, a deformed right femoral head with postpinning defect, articular changes, and subluxation was visualized. After initial resuscitation, exploratory laparotomy was performed immediately with subsequent ligation of feeding vessel to the pseudoaneurysm and appendectomy (Figures 4 and 5).

**Figure 6:**
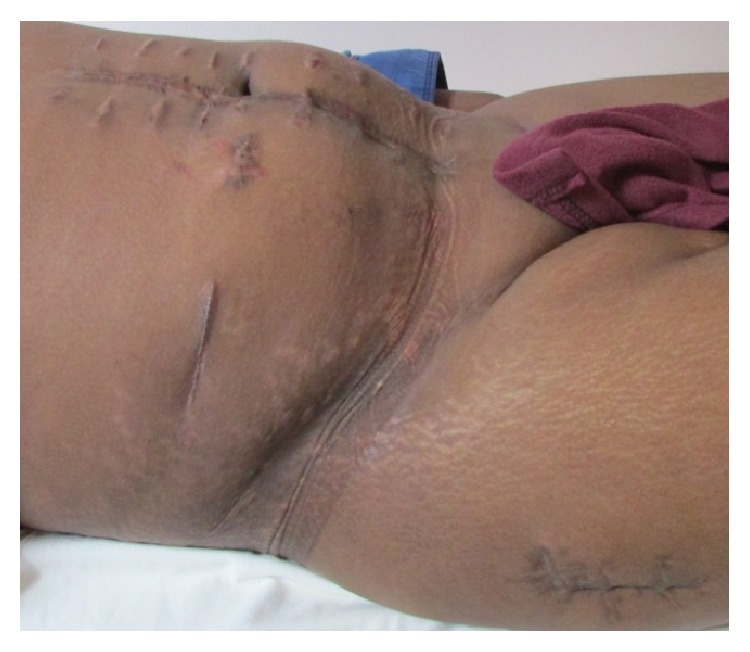
Healed, healthy scars of postoperative wounds at 6-month follow-up.

**Figure 7:**
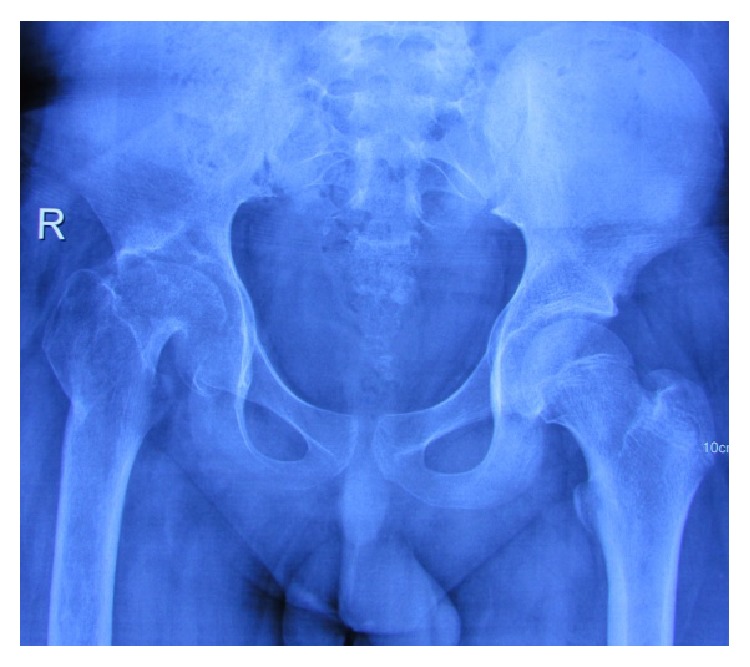
Radiograph of hip joint at 6-month follow-up.

Days after surgery the patient was recovering well and his rectal bleeding stopped. However, his condition started deteriorating again with worsening of general condition, high grade fever, and other features of septicemia. Ultrasound abdomen revealed heterogeneous collections in the right medial thigh and abdomen, which suggested infection of the remaining hematomas. Exploratory laparotomy and exploration of medial thigh were done. Around 2 liters of pus was drained intraoperatively from both wounds and a drain was placed in situ. The condition of the patient improved gradually and the patient was discharged after 2 months on crutches. On follow-up after six months, the patient was walking comfortably without any aid and he has returned to his day to day activities. The surgical wounds have healed and scars appear healthy (Figure 6). Radiograph at follow-up shows deformed femoral head on the right side (Figure 7). True shortening of 2 cm of the right lower limb was found on follow-up, but no surgical intervention was done since the patient did not have any complaints.

## 3. Discussion

Two types of K-wires are used most commonly: threaded and smooth [1]. Complications related to migration are more seen with smooth than threaded K-wires. Usually the migration of K-wire is retrograde along the path of its insertion since this is the path of least resistance due to muscular movement and the smooth nature of the K-wire [2]. Antegrade migration of K-wire, though rare, has been reported in the literature. Several case reports have been reported on the migration of K-wire from shoulder joint to various intrathoracic organs such as aorta [3], heart [4, 5], lungs [6, 7], trachea [8, 9], mediastinum [10], neck [11], spleen [12], and spinal canal [13, 14]. In two of the cases migration of K-wires led to death of the patient [4, 6]. Few cases of migration from hip to heart [15, 16], liver [2], and popliteal fossa [17] have been reported. A single case of K-wire migration from left hand to the heart has also been reported [18]. In one of the cases where the K-wire migrated from hip to liver [2], there was no injury to the intervening abdominal structures in contrast to our case. Vascular injuries related to K-wire insertion have been reported in upper limb [3] but never in lower limbs. In a case reported by Rossi et al. [17], a K-wire migrated from hip to popliteal fossa, but no vascular injury resulted and the wire was found between semimembranosus and semitendinosus muscle. Thus, our case represents the first case report of K-wire migration which caused vascular injury and resulted in life-threatening injury to the patient. In addition, our case describes late onset migration after several months due to the gradual migration of K-wire.

Most of the complications of K-wire migration result in minor injuries. There are several articles citing migration from proximal humeral and clavicular fracture fixation resulting in intrathoracic vascular injury [3, 19, 20]. Almost all injuries result from smooth K-wire insertion. Moreover, the sequelae due to migration, as seen in our case, have never been reported. Similar to other cases, our reported case of migration involved injury with smooth K-wire. The reason for migration in our case was lack of anchorage to surrounding structure due to the smooth nature of the wire along with technical fault of insertion such as not bending the end of the wire and burying the K-wire under the skin. These factors likely facilitated antegrade migration due to pressure on the K-wires during the times the patient lay down on the same side of insertion in addition to muscular propulsion. Unlike the other described cases of intra-abdominal migration where no significant injury occurred, there was a life-threatening complication in our case leading to vascular injury and subsequent intra-abdominal and medial thigh compartment abscess formation along with septicemic shock necessitating numerous surgeries resulting in significant morbidity. Had it not been for timely intervention, the death of the patient would have been inevitable.

## 4. Conclusion

Our case demonstrates that antegrade intra-abdominal migration of K-wire from hip joint may lead to life-threatening injuries. Caution should be exercised when inserting a smooth K-wire. Complications related to migration of K-wire can be minimized by bending the end of the K-wire, by keeping the tip protruding outside the skin wherever possible, and by early removal of K-wire once it is no longer needed.
